# The Promising Role of a Superb Microvascular Imaging Technique in the Evaluation of Raynaud’s Syndrome in Systemic Sclerosis: Theory and Practical Challenges

**DOI:** 10.3390/diagnostics11101743

**Published:** 2021-09-22

**Authors:** Gabija Jasionyte, Goda Seskute, Rita Rugiene, Irena Butrimiene

**Affiliations:** 1 Clinic of Rheumatology, Orthopaedics Traumatology and Reconstructive Surgery, Institute of Clinical Medicine, Faculty of Medicine, Vilnius University, LT-03101 Vilnius, Lithuania; goda.seskute@santa.lt (G.S.); rita.rugiene@santa.lt (R.R.); irena.butrimiene@santa.lt (I.B.); 2 Department of Experimental, Preventive and Clinical Medicine, State Research Institute Centre for Innovative Medicine, LT-08406 Vilnius, Lithuania

**Keywords:** Doppler, nailfold capillaroscopy, superb microvascular imaging, ultrasound

## Abstract

In recent years, a novel Doppler ultrasonography (US) modality—superb microvascular imaging (SMI)—has been presented as a reliable method to evaluate small vessel blood flow with minimised motion artefacts. In this review, we present the challenges of incorporating SMI in daily practice with detailed and comparable US images of a fingertip. The main focus of this paper is the discussion of all tested US techniques, artefacts, and practical challenges for evaluating Raynaud’s syndrome in systemic sclerosis. Despite a few reports on SMI use in assessing nailfold capillaries, there is still a need for more evidence of its value and possibilities for its standardisation.

## 1. Introduction

New emerging diagnostic techniques have made a dramatic breakthrough in the diagnostics and management of rheumatic diseases. First introduced in 1973 by Maricq, today, nailfold videocapillaroscopy (NVC) is a routinely used diagnostic method [1]. This non-invasive and easily performed instrumental tool enables one to differentiate primary from secondary Raynaud‘s phenomenon [2]. The abnormality of nailfold capillaries is among the 2013 ACR/EULAR classification criteria for systemic sclerosis (SSc) [3]. Furthermore, NVC can lead to an early diagnosis of SSc, since nailfold microvascular alterations appear in an early stage of the disease [4]. Specific NVC patterns can reflect alterations in the whole body and predict the severity of organ involvement [5,6]. Apart from diagnostic advantages, NVC might be useful in predicting the risk of new digital ulcer formation in SSc or evaluating the clinical effect of treatment [7,8].

Ultrasonography (US) has been integrated into rheumatologists’ practices even more extensively. It can be used to detect various pathological changes, such as synovial inflammation, enthesopathy, tendinopathy, bursitis, and monitor disease progression or guide articular aspiration or injection [9]. Some authors have presented the advantages of ultrasonography in evaluating digital vascularisation. In 2000, Keberle et al. were the first to report that assessing nailfold capillaries with colour Doppler ultrasonography helps to reliably discriminate between primary and secondary Raynaud’s phenomenon (RP) [10]. Other authors used power Doppler (PD) ultrasound to evaluate nailfold vascularity, and found it to be a promising method to diagnose RP [11]. Kim et al. later reported that PD might be even more accurate than NVC in differentiating between these two conditions [12]. In 2018, Flower et al. presented a high-frequency ultrasound with superb microvascular imaging (SMI) as a novel measure to assess microvascular abnormalities. They reported that this modality allowed the evaluation of a wider spectrum of broad regions of interest (ROI), including the nailfold, and with minimised motion artefacts [13].

**Figure 1:**
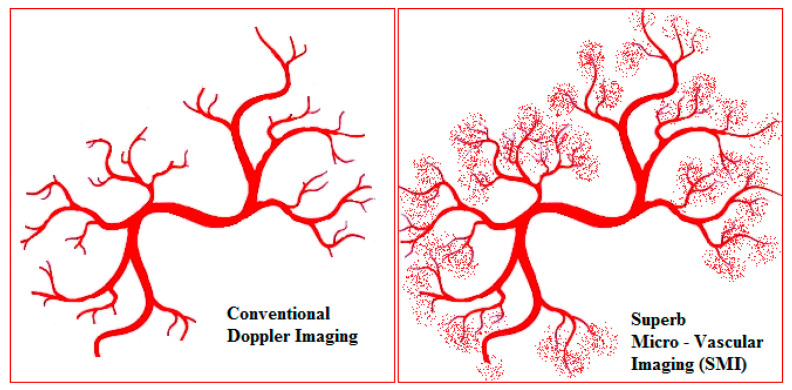
The schematic view of the difference between power Doppler and superb microvascular imaging (SMI) in the evaluation of small low-flow vessels.

SMI is a novel Doppler modality that provides the visualisation of microvascular flow never detected before by US. It can suppress the noise caused by motion artefacts with an innovative filter system without removing the weak signal arising from small vessel blood flow (Figure 1). SMI presents two modes: colour (cSMI, which demonstrates B-mode and colour information simultaneously) and monochrome (mSMI, which focuses only on the vasculature) [14]. Both modes demonstrate the value of differentiating a wide variety of clinical situations: inflammatory diseases (arthritis, colitis), tumours, and the therapeutic effect of a treatment [15,16]. In recent years, the role of high-frequency ultrasound for the evaluation of digital microvascularity in patients with RP has grown, but the value of US in daily fingertip (especially nailfold zones) investigation remains debatable given its lack of evidence.

We aimed to review and report our first experience and technique of using a high-frequency ultrasound with an SMI technique for assessing digital vasculopathy in SSc.

## 2. Vascular Anatomy of the Fingertip

**Figure 2:**
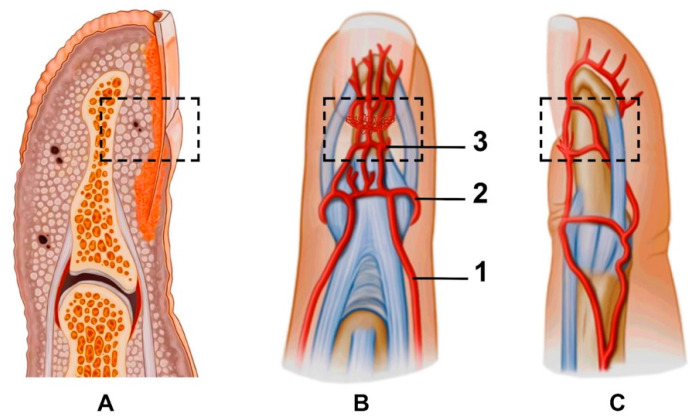
(**A**) Macroanatomy of the fingertip; anterior (**B**) and lateral (**C**) views of the fingertip and nailfold vasculature: 1—proper palmar digital artery, 2—superficial arcade, 3—proximal subungual arcade. Branches supplying the fingertip pulp start from the distal subungual arcade. Dotted boxes mark the anatomical zone of the proximal nailfold vascularity.

The fingertip is defined as the part of the digit distal to the flexor and extensor tendons’ insertion into the distal phalanx (Figure 2A). The diameter of the vessels and the wall thickness decrease as we proceed distally, but the vessels are much larger than one might imagine and are easily found under magnification [17]. The arterial blood supply of the fingertip arises from two proper palmar digital arteries joining to the superficial arcade at the base of the distal phalanx, also referred to as the “dorsal nailfold arch”. The superficial arcade perfuses the dorsal skin of the fingertip and the nail complex [18,19]. The longitudinal branches of the superficial arcade run deep to the germinal matrix and nail root to form the proximal subungual arcade. The proximal subungual arcade gives off some longitudinal branches distally, which then unite as the next transverse arcade, which is called the distal subungual arcade (Figure 2B,C). 

When performing NVC, the target is multiple superficial branches from the superficial arcade in the proximal and lateral nailfold zones. An ultrasound expands the possibility of seeing vascularity under the nail plate in the dorsal volar nailfold (involves the proximal subungual arcade and its branches) and fingertip pulp (the branches from the distal subungual arcade). 

## 3. Technical Aspects of the Fingertip Ultrasound Imaging

**Figure 3:**
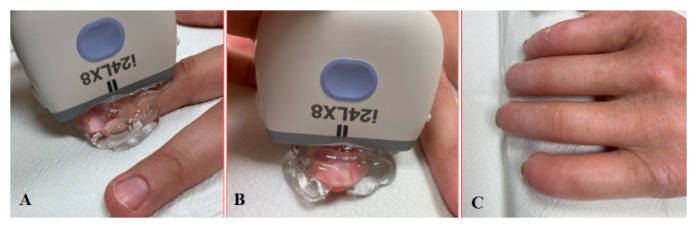
Longitudinal (**A**) and sagittal (**B**) positions during the US investigation. (**C**) Comfortable position for patients with sclerodactyly allows us to make high-quality images and avoid artefacts.

The patient undergoing nailfold ultrasound is seated facing the investigator. The wrist and freely spread fingers are placed in a neutral position on an adjustable stand (Figure 3A,B). The examinations have to be performed after 10–15 min of acclimation in a room with an ambient temperature. The first problem we noticed was limited finger extension due to skin thickening, especially in the late course of the disease. In this case, the option is to put the roller under the hand then the patient can relax the fingers comfortably without small movements (Figure 3C). The mound of gel facilitates proper transducer orientation without pushing the nailfold, and it helps to avoid random noise or motion artefacts. The warm gel prevents a Raynaud’s syndrome attack during the investigation. Lee et al. evaluated nailfold microvascularity using PD before and after placing the hand into cold water 7 °C for 3 min [11]. US examination with a cold challenge could be suitable only for research purposes because provocation of RP causes discomfort or even pain for the patient and takes too much time for daily investigations.

We think that examinations should be performed and interpreted by an experienced investigator, since US, especially the SMI modality, is an operator-dependent and sensitive method. The investigator should be familiar with its technical aspects and able to recognise artefacts and differentiate them from significant alterations.

## 4. PD and SMI Findings in the Nailfold

**Figure 4:**
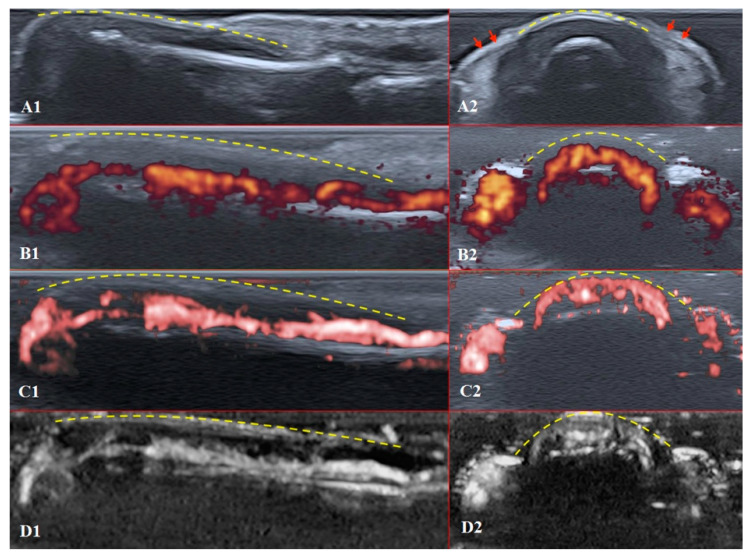
Ultrasound images of the healthy fingertip of the second finger of a hand: (**A1**,**A2**) B mode/grayscale; (**B1**,**B2**) PD; (**C1**,**C2**) cSMI; (**D1**,**D2**) mSMI. The yellow dotted lines mark the nail. Sagittal scans (**B1**–**D1**) in the midline of the fingertip show vascularity from the deep layers of the nailbed to the end of fingertip pulp. The main landmarks for all transverse scans (**B2**–**D2**) are lateral nailfolds (**A2** red arrows), which unite in the middle of the proximal nailfold and become a convex line.

**Figure 5:**
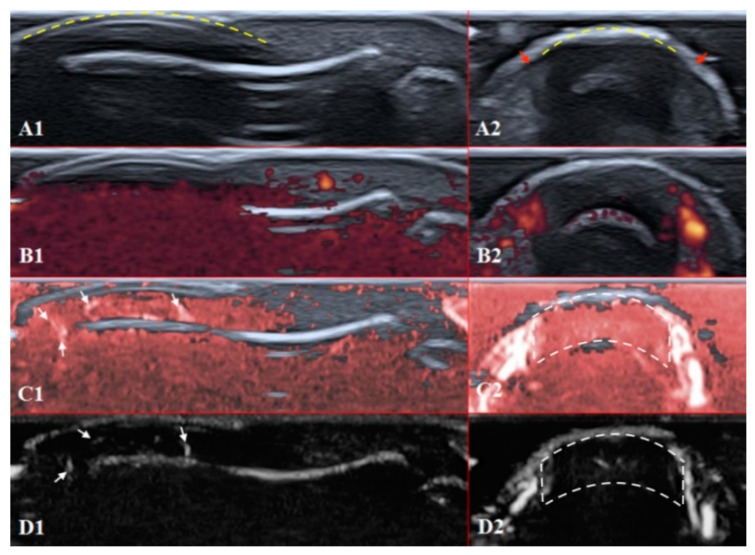
Ultrasound images of the nailfold of the fifth finger of the patient with a late course of SSc: (**A1**,**A2**) B mode/grayscale; yellow dotted lines mark the nail, red arrows mark the lateral nailfold, which is decreased due to sclerodactyly. (**B1**,**B2**) PD; there is an avascular zone in B2 in the deeper layers of the nailfold; B1 does not show any vascular sign with maximal gain. (**C1**,**C2**) cSMI; white arrows mark the true flow in the random noise background; the area marked by dotted lines in C2 is also avascular and the true flow signals on the sides show the lateral nailfold. (**D1**,**D2**) mSMI; confirms cSMI findings.

The structures of the nailfold are too small to assess by a conventional US. Thus, adequate sonographic evaluation is dependent on high-resolution techniques and high-frequency linear array probes. We used a diagnostic ultrasound system (CANON TUS-AI800, Canon medical systems Corp., Shimoishigami, Otawara-shi, JAPAN) equipped with a linear transducer with the following settings: 24 MHz ultrahigh-frequency (Canon medical systems Corp., Shimoishigami, Otawara-shi, JAPAN). Sagittal (dorsal volar) and transverse scans of fingertips were performed on a healthy volunteer (Figure 4) and a patient with late-course SSc (Figure 5). ROI was located between the fingernail and the bony surface of the distal phalanx. Up-to-date versions of SMI equipment provide a direct-control scale function. Therefore, the investigators do not need to worry about ROI.

**Figure 6:**
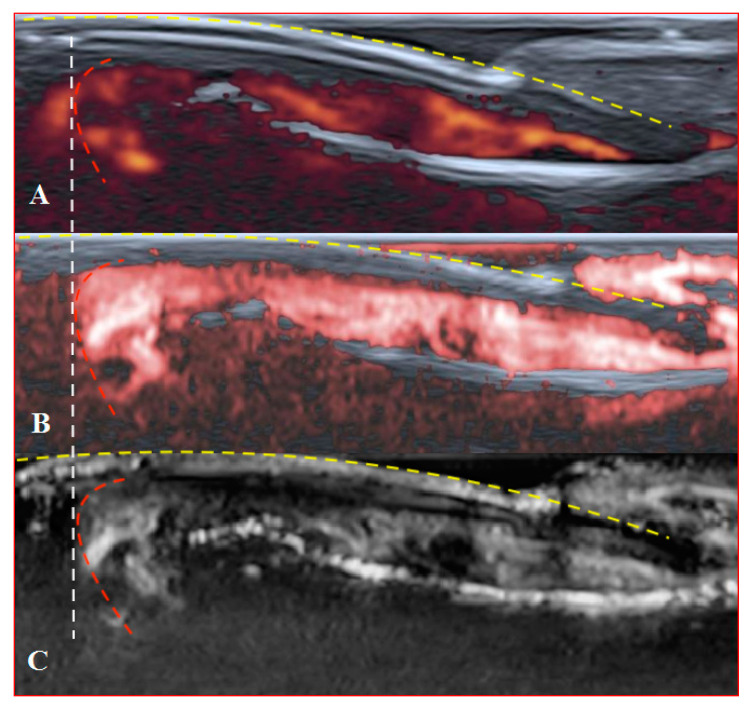
Sagittal scans of a healthy fingertip with high gain: (**A**) PD; (**B**) cSMI; (**C**) mSMI. The true flow is the highest signal that dissociates from the random noise dots clearly. It is hard to say how much blooming artefact is involved. The transverse dotted line through all images separates the area under the nail close to the fingertip (red dotted line); a gel manicure does not change the quality of the image but longer nails provoke flash artefacts.

**Figure 7:**
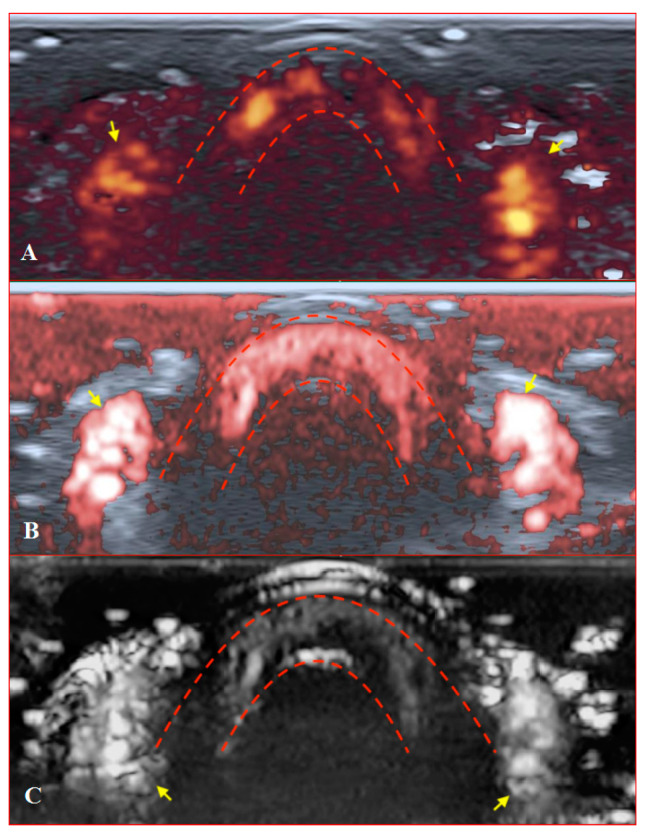
Transverse scans of a healthy fingertip with high gain: (**A**) PD; (**B**) cSMI; (**C**) mSMI. Red dotted lines define the zone between the nail and the surface of a distal phalanx. Yellow arrows mark the lateral nailfold.

PD and SMI settings have to be standardised for all evaluations. There is an appropriate option to differentiate primary Raynaud’s syndrome from its secondary development by setting the gain. Martinoli et al. suggested increasing the gain maximally and then slowly lowering it until the noise disappears and true signals remain [20]. Rubin offered a converse way to raise the gain manually until the colour box becomes filled with signals and a true flow is distinguished from the background as the next highest signal [21]. The method by Rubin seems to be a quick and comfortable technique to check vascularity in fingertip pulp and nailfold zones (Figure 6 and Figure 7). It takes less time to evaluate vascularity by SMI modes rather than the conventional PD technique due to its higher sensitivity for low flow. Monochrome SMI is a comfortable mode for the evaluation of vasculature and even vascular torsions, as the true flow has more power, and it is easier to separate these dots from the background of random noise artefacts (Figure 4D2). 

The difference between healthy and SSc-affected fingertips is obvious using Rubin’s method. Higher gain shows that there is no signal in PD and minimal dots with both SMI modes (Figure 5).

## 5. Discussion

The following capillaroscopic characteristics are evaluated in a standardised manner when assessing a capillaroscopic image: capillary density, capillary dimension, presence/absence of abnormal shapes, and presence/absence of haemorrhages [22]. In the case of SSc, the “scleroderma pattern” may be present which is graded into “early”, “active”, or “late” patterns according to the found characteristics. No significant differences were identified by SMI or any other Doppler techniques between SSc capillaroscopic classifications [13].

The results of nailfold area ultrasound depend on the investigation target and measurement (quantitative or qualitative) choice:

1. Arterioles of the nailfold. Doppler spectral analysis at the level of a distal nailfold arteriole was used to measure and compare parameters such as proximal resistive index (RI) and peak systolic velocity (PV) among healthy subjects and patients with SSc. Lower PV and higher distal RI were found in patients with SSc [23]. Schioppo et al. calculated RI at wider diameter vessels—radial and ulnar proper digital arteries at the proximal phalanx level. Checking for ulnar artery occlusion (UAO) before the assessment is recommended [24].
2. ROI at the area of the fingertip (dorsovolar, nailfold, and fingertip pulp). cSMI showed significantly reduced vascularity indices at these regions in SSc with each of the capillaroscopic SSc patterns [13]. A specialised software enabled Keberle et al. to obtain the absolute number of colour signals in ROI and define the nailfold perfusion quantitatively [10]. However, most authors [11,12,24] describe the vascularity of nailfold zones qualitatively and grade it on a scale from 1 (no signal) to 4 (marked hyperemia), as suggested by Newman et al. [25].

The qualitative evaluation of the nailfold zone is more popular among researchers because it is easier to interpret and compare the results. Measurement of indices has a value regarding the comparison of treatment effects. Correlations of US results with other tools represent another opportunity to ascertain SSc pattern differentiation. Lee et al. reported a strong correlation between the findings obtained by PD and NVC in differentiating primary from secondary RP [11]. Other authors found that nailfold US differentiates RP with better accuracy than NVC [12]. In addition, a good correlation between capillary number at NVC and nailfold perfusion assessed by PD was reported [24]. On the other hand, Freire et al. found no association between a particular NVC pattern and nailfold PD findings in patients with SSc [26]. The possibility of distinguishing SSc patterns remains questionable.

Doppler settings play an important role in avoiding artefacts, especially when evaluating small structures of the fingertip zone. Due to a lack of time, adjusting all Doppler parameters is not performed at every examination. The most common artefact performing the US is random noise because it is detected each time during the investigation until parameters are adjusted. Optimal gain (depends on using Rubin’s method), adequate pressure on the transducer with a mound of warm gel, and an appropriate fingertip positioning with complete tissue relaxation are the most important tips for exploring fingertip zones by all Doppler modalities [22]. 

The first steps for using ultrasound in nailfold evaluation are growing and promising. In 2018, CANON Medical created a 33 MHz Ultra-High frequency transducer. Its frequency is the highest among all transducers accessible in medical practice. For this reason, it might be a promising tool for superficial subcutaneous imaging. This promising progress will lead to the evaluation of superficial vascularity, including nailfold and nailbed (inner layers) and damaged skin layers at the beginning of the formation of ulcers. We should expect new reports with highly detailed images of the nailfold shortly.

In conclusion, SMI seems to be a promising method in the evaluation of microvascular damage in the nailfold. Despite some intriguing findings from a few authors, there are still few reliable data about the full potential of SMI in the evaluation of microvasculature. More evidence from larger, well-designed studies, especially comparing SMI with other validated methods such as NVC, power Doppler US, and/or Laser speckle contrast analysis (LASCA), is needed. A new challenge could be checking the relation between nailfold vascularity alterations and the severity of larger vessel damage by high-end ultrasound modalities. The goal is to find the best standardised technique for research and daily clinical practice.

## Data Availability

Not applicable.
